# A systematic review of the validated monogenic causes of human male infertility: 2020 update and a discussion of emerging gene–disease relationships

**DOI:** 10.1093/humupd/dmab030

**Published:** 2021-09-08

**Authors:** Brendan J Houston, Antoni Riera-Escamilla, Margot J Wyrwoll, Albert Salas-Huetos, Miguel J Xavier, Liina Nagirnaja, Corinna Friedrich, Don F Conrad, Kenneth I Aston, Csilla Krausz, Frank Tüttelmann, Moira K O’Bryan, Joris A Veltman, Manon S Oud

**Affiliations:** 1 School of BioSciences and Bio21 Institute, The University of Melbourne, Parkville, VIC, Australia; 2 Andrology Department, Fundació Puigvert, Universitat Autònoma de Barcelona, Instituto de Investigaciones Biomédicas Sant Pau (IIB-Sant Pau), Barcelona, Catalonia, Spain; 3 Institute of Reproductive Genetics, University of Münster, Münster, Germany; 4 Andrology and IVF Laboratory, Division of Urology, Department of Surgery, University of Utah School of Medicine, Salt Lake City, UT, USA; 5 Department of Nutrition, Harvard T.H. Chan School of Public Health, Harvard University, Boston, MA, USA; 6 Faculty of Medical Sciences, Biosciences Institute, Newcastle University, Newcastle upon Tyne, UK; 7 Division of Genetics, Oregon National Primate Research Center, Oregon Health & Science University, Beaverton, OR, USA; 8 Genetics of Male Infertility Initiative (GEMINI); 9 International Male Infertility Genomics Consortium (IMIGC); 10 Department of Experimental and Clinical Biomedical Sciences “Mario Serio”, Centre of Excellence DeNothe, University of Florence, Florence, Italy; 11 Department of Human Genetics, Donders Institute for Brain, Cognition and Behaviour, Radboudumc, Nijmegen, the Netherlands

**Keywords:** genetics, male infertility, clinical validity, gene panel, gene–disease relationship, next-generation sequencing, spermatogenic failure, multiple morphological abnormalities of the sperm flagella, systematic review

## Abstract

**BACKGROUND:**

Human male infertility has a notable genetic component, including well-established diagnoses such as Klinefelter syndrome, Y-chromosome microdeletions and monogenic causes. Approximately 4% of all infertile men are now diagnosed with a genetic cause, but a majority (60–70%) remain without a clear diagnosis and are classified as unexplained. This is likely in large part due to a delay in the field adopting next-generation sequencing (NGS) technologies, and the absence of clear statements from field leaders as to what constitutes a validated cause of human male infertility (the current paper aims to address this). Fortunately, there has been a significant increase in the number of male infertility NGS studies. These have revealed a considerable number of novel gene–disease relationships (GDRs), which each require stringent assessment to validate the strength of genotype–phenotype associations. To definitively assess which of these GDRs are clinically relevant, the International Male Infertility Genomics Consortium (IMIGC) has identified the need for a systematic review and a comprehensive overview of known male infertility genes and an assessment of the evidence for reported GDRs.

**OBJECTIVE AND RATIONALE:**

In 2019, the first standardised clinical validity assessment of monogenic causes of male infertility was published. Here, we provide a comprehensive update of the subsequent 1.5 years, employing the joint expertise of the IMIGC to systematically evaluate all available evidence (as of 1 July 2020) for monogenic causes of isolated or syndromic male infertility, endocrine disorders or reproductive system abnormalities affecting the male sex organs. In addition, we systematically assessed the evidence for all previously reported possible monogenic causes of male infertility, using a framework designed for a more appropriate clinical interpretation of disease genes.

**SEARCH METHODS:**

We performed a literature search according to the PRISMA guidelines up until 1 July 2020 for publications in English, using search terms related to ‘male infertility’ in combination with the word ‘genetics’ in PubMed. Next, the quality and the extent of all evidence supporting selected genes were assessed using an established and standardised scoring method. We assessed the experimental quality, patient phenotype assessment and functional evidence based on gene expression, mutant *in-vitro* cell and *in-vivo* animal model phenotypes. A final score was used to determine the clinical validity of each GDR, across the following five categories: no evidence, limited, moderate, strong or definitive. Variants were also reclassified according to the American College of Medical Genetics and Genomics-Association for Molecular Pathology (ACMG-AMP) guidelines and were recorded in spreadsheets for each GDR, which are available at imigc.org.

**OUTCOMES:**

The primary outcome of this review was an overview of all known GDRs for monogenic causes of human male infertility and their clinical validity. We identified a total of 120 genes that were moderately, strongly or definitively linked to 104 infertility phenotypes.

**WIDER IMPLICATIONS:**

Our systematic review curates all currently available evidence to reveal the strength of GDRs in male infertility. The existing guidelines for genetic testing in male infertility cases are based on studies published 25 years ago, and an update is far overdue. The identification of 104 high-probability ‘human male infertility genes’ is a 33% increase from the number identified in 2019. The insights generated in the current review will provide the impetus for an update of existing guidelines, will inform novel evidence-based genetic testing strategies used in clinics, and will identify gaps in our knowledge of male infertility genetics. We discuss the relevant international guidelines regarding research related to gene discovery and provide specific recommendations to the field of male infertility. Based on our findings, the IMIGC consortium recommend several updates to the genetic testing standards currently employed in the field of human male infertility, most important being the adoption of exome sequencing, or at least sequencing of the genes validated in this study, and expanding the patient groups for which genetic testing is recommended.

## Introduction

Male infertility is a common condition, affecting at least 7% of men worldwide, and is often predicted to be largely genetic in origin (Krausz and Riera-Escamilla, 2018). A majority of all human (84%, 16 598; Uhlen *et al.*, 2015) and mouse (90%, 18 037; Schultz *et al.*, 2003) protein-coding genes are expressed in the testis, emphasising that sperm production is a complex process and involves many separate biological pathways. Furthermore, sperm must mature in the epididymis and undergo a final step of activation in the female reproductive tract, termed ‘capacitation’, before they are capable of fertilising an oocyte. By extension, there are many points during sperm development and maturation that could be compromised by the effect of genetic variants. The identification of genes affected by these variants and the assessment of definitive genotype–phenotype correlations, however, remains a challenge.

Advances in next-generation sequencing (NGS) have greatly facilitated the unbiased exome-wide (whole-exome sequencing (WES)) and genome-wide (whole-genome sequencing (WGS)) detection of any genetic variants that may play a role in male infertility (e.g. Coutton *et al*., 2019; Alhathal *et al.*, 2020; Chen *et al.*, 2020; Liu *et al.*, 2021). However, the population frequency of these variants is expected to be very low given their negative effect on fertility. For this reason, the analysis of large patient and control cohorts is essential to identify recurrently mutated genes and detect statistical enrichments in patient cohorts. One major problem in the male infertility field is that genetic testing strategies employed in andrology clinics are not standardised, and in some countries/states even the most basic of tests (karyotype and azoospermia factor deletion analysis) are still not routinely used.

The male infertility field is currently catching up with other fields/disease types with a strong genetic component, such as intellectual disability, neuromuscular disorders and hereditary hearing impairments (Chiu *et al.*, 2020; Markitantova and Simirskii, 2020; Whatley *et al.*, 2020). The rapid uptake of NGS technologies in the male infertility research field over the past 5–10 years, as well as the development of international consortia to collect and characterise clinical cohorts, is aiding in the transition of findings into clinical practice. To assist in this feedback of knowledge, clear direction is required for the validity of which individual genes to be screened and their relevance to certain types of infertility.

In this article, we provide an updated clinical validity assessment of the monogenic causes of male infertility (Oud *et al.*, 2019), through the systematic analysis of newly published evidence from January 2019 to July 2020 for individual gene–disease relationships (GDRs). We have employed the joint expertise of the International Male Infertility Genomics Consortium (IMIGC) to systematically evaluate/re-evaluate all available evidence for published monogenic causes of isolated or syndromic male infertility, endocrine disorders that impact male fertility and reproductive system abnormalities affecting the male sex organs. This analysis has resulted in the identification of 104 high-probability ‘human male infertility genes’, a 33% increase from the number identified in 2019.

## Methods

### Search strategy and study selection

A literature search was performed as described in Oud *et al.* (2019) to identify articles reporting on monogenic causes of male infertility or male reproductive system anomalies entered into MEDLINE-PubMed before 1 July 2020. Assessment of whether the articles met the inclusion or exclusion criteria (detailed in Supplementary Table SI) was performed by two independent reviewers (B.J.H. and M.S.O.). The present study and the corresponding search protocol were registered with the PROSPERO registry (http://www.crd.york.ac.uk/PROSPERO) as PROSPERO 2021: CRD42021229164.

### Data extraction and assessment

The clinical validity of each identified GDR was scored using a system published by Smith *et al.* (2017). Scoring was performed by two reviewers, randomly assigned from the pool of eight reviewing authors (B.J.H., A.R.-E., M.J.W., A.S.-H., M.J.X., L.N., C.F. and M.S.O.) using a standardised assessment template to extract gene names, inheritance patterns, patient phenotypes, method of discovery (sequencing method); annotate variants; and assess both functional evidence and clinical data, including the outcome of ART and recorded comorbidities. Expression of genes across human organs was assessed by consulting data available on the Human Protein Atlas website, NCBI’s RNA-seq dataset (Fagerberg *et al.*, 2014), GTEx and an unpublished human testis single-cell RNA-seq library tool (https://conradlab.shinyapps.io/HISTA). To avoid bias in gene–disease evaluation and any conflicts of interest, reviewers were not allowed to score any GDRs they had published. Scoring was separated into five categories: no evidence (<3 points), limited (3–8 points), moderate (9–12 points), strong (13–15 points) or definitive (>15 points), with a maximum of 17 points. After independent scoring, the individual scores (for each GDR) for both reviewers were compared and any inconsistencies in scoring (>1 point difference or a difference in the final classification) were settled by the two assigned reviewers. Where this was not possible, the scoring was discussed with all non-conflicted reviewers. GDRs with a moderate or higher classification were deemed as confidently linked to human male infertility and combined scoring sheets are available at http://www.imigc.org. All results in the study are collated in Supplementary Table SII.

## Results

### Summary of included studies and design

**Figure 1.**
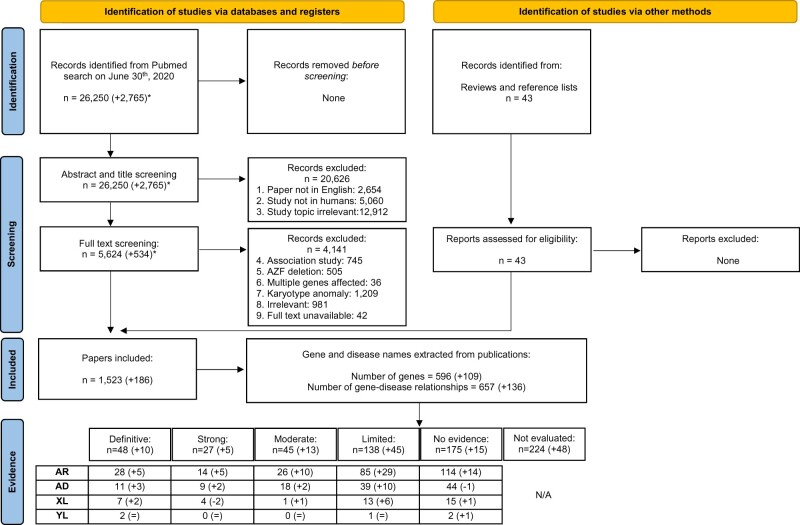
**PRISMA flowchart of search and assessment process.** AZF, azoospermia factor; R, autosomal recessive; AD, autosomal dominant; XL, X-linked; YL, Y-linked.

**Figure 2.**
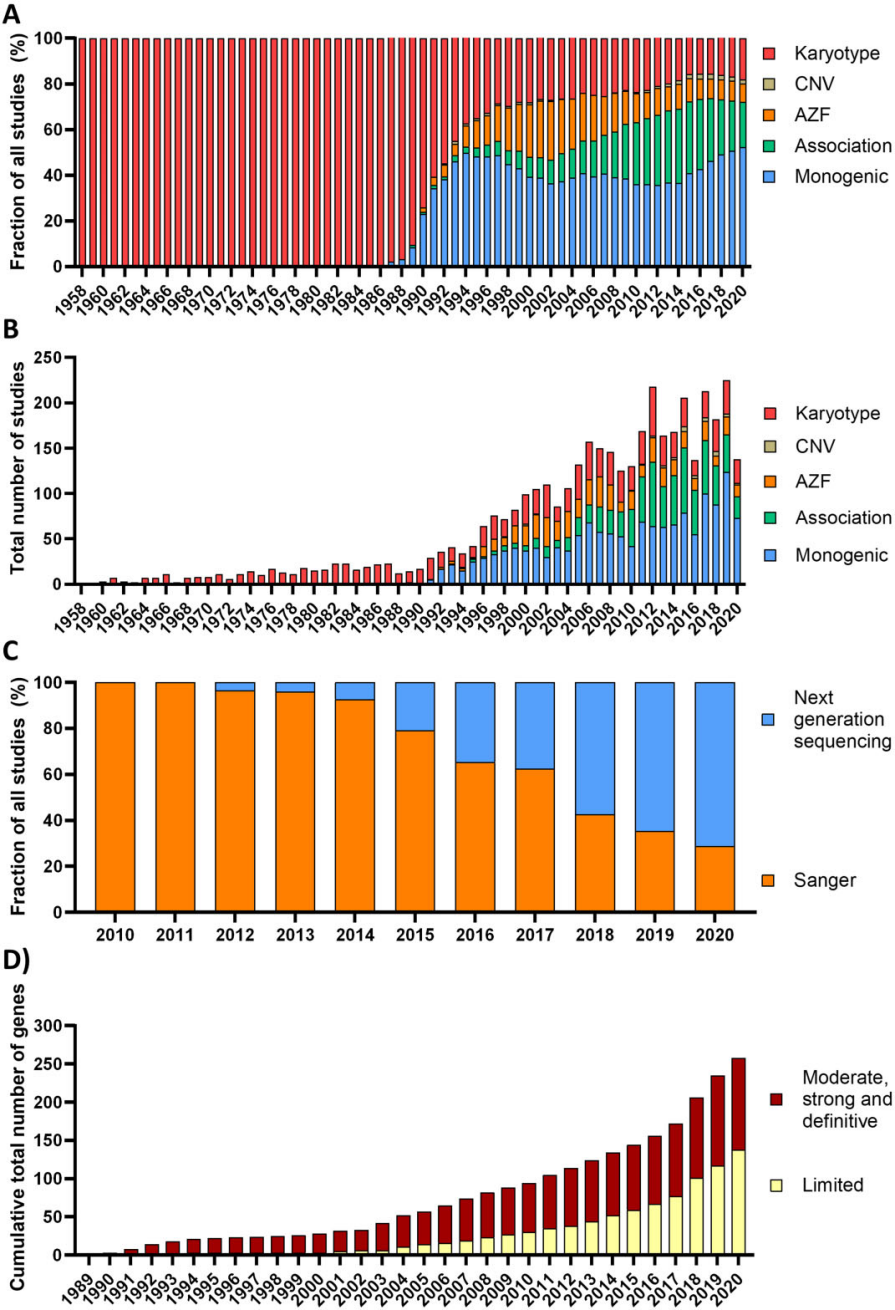
**Breakdown of genetic testing approach and number of genes associated with male infertility phenotypes with limited or moderate and higher evidence classification, over time.** (**A**) The fraction of studies assessed in this paper that used particular sequencing technologies to elucidate causes of male infertility from 1958 to 2020, including karyotyping, copy number variation assessment, azoospermia factor region deletion assessment, association studies and studies investigating monogenic causes. (**B**) The total number of studies assessed in this paper investigating male infertility, from 1958 to 2020, based on sequencing approach. (**C**) The fraction of studies assessed in this paper using next-generation sequencing technology compared to Sanger sequencing, from 2010 to 2020. (**D**) The cumulative number of genes and their strength of evidence as linked to male infertility phenotypes based the scoring criteria used in this paper, from 1989 to 2020. CNV, copy number variant.

We performed a literature search using terms related to ‘male infertility’ in combination with keywords related to the word ‘genetics’ in MEDLINE-PubMed and used the same inclusion and exclusion criteria as described previously (Oud *et al.*, 2019). A total of 26 250 articles were identified, of which 2765 new articles were identified since the previous search in 2018 and not covered by the previous review (Fig.  1). The total number of publications on genetic causes of male infertility has increased by 13% from 849 over the period 2010–2014 to 963 articles in the period 2015–2019 (Fig.  2A). Furthermore, while the final search performed for this review was completed in mid-2020, the total number of 2020 studies was estimated to overtake the 2019 number by 20%. The absolute and relative contribution of publications on proposed monogenic causes of phenotypes related to male infertility is also growing (46% in 2017 versus 52% in 2020). The relative contribution of publications on association studies has declined by 42% (27% in 2017 versus 19% in 2020; Fig.  2B). The shift away from Sanger sequencing to NGS methods is continuing to take place, and 71% of all sequencing studies used NGS in 2020 (Fig.  2C), which constitutes an increase of 90% since 2017. While WGS has started to emerge in the field (recent examples: Bedoni *et al.*, 2016; Wang et al., 2017; Arafat *et al.*, 2020; Yuan *et al.*, 2020), WES is currently still the predominant method over panel sequencing and WGS (75% versus 19% and 6%, respectively). Nevertheless, there are clinical presentations where Sanger/panel sequencing is still being utilised at a high frequency, e.g. 63% of studies on 46,XX and 46,XY disorders/differences of sexual development (DSDs) in the period of 2019–2020.

### Systematic evaluation of evidence

A total of 1523 publications met all inclusion criteria and were used in this systematic review. Of these, 186 publications were published in the period from 2019 to mid-2020 and were thus not included in the previous clinical validity assessment. Overall, 657 individual GDRs described in these 1523 publications were investigated, of which 136 were novel and 521 were re-evaluated with our updated assessment criteria in order to incorporate any additional supporting evidence (Supplementary Table SII).

The quality and the extent of all evidence for the GDRs were assessed by two independent reviewers, using a standardised scoring method. This assessment was performed for GDRs that were new (n = 136), existing with newly published evidence (n = 145) or previously confidently linked (moderate, strong or definitive) to male infertility with no newly published evidence (n = 19). This score was used to assign a clinical validity of each GDR as: no evidence, limited, moderate, strong or definitive. We assessed the experimental quality, patient phenotype information, functional evidence based on gene expression and the study of *in-vitro and in-vivo* loss-of-function animal/cell models. Variants were also reclassified according to the widely accepted the American College of Medical Genetics and Genomics-Association for Molecular Pathology (ACMG-AMP) standards and then recorded in spreadsheets for each GDR.

Of the newly identified GDRs, 17 were classified as having moderate evidence or higher (confidently associated with the phenotype) and 43 were classified as having limited evidence. After classification of the existing GDRs that had new evidence published and scoring of GDRs that were previously confidently linked to male infertility with our updated scoring criteria, 45 increased in score since 2019, 11 decreased in score and 9 changed from unable to be classified to no evidence (or *vice versa*). As detailed above, GDRs were listed as ‘unable to classify’ when the predicted inheritance pattern or genotypes were not reported. In comparison to the previous assessment, 456 scores did not change because no (or insufficient) new evidence was published during the search period (Supplementary Table SII).

**Figure 3.**
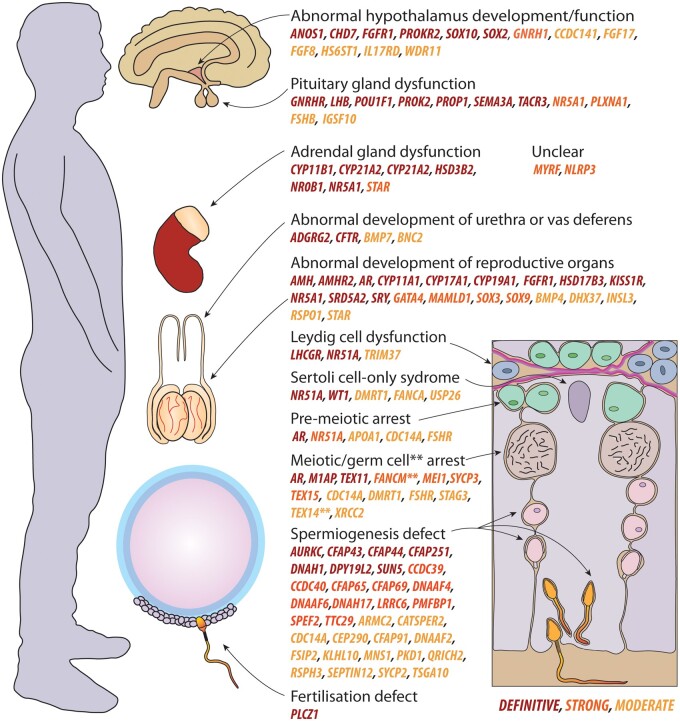
**Overview of all genes associated with male infertility phenotypes at an organ/cell level.** Definitive genes are labelled in red, strong in orange and moderate in yellow. Organs top to bottom: brain, adrenal gland (and kidney), testes and epididymides with vas deferens. Bottom left: sperm fertilising an oocyte surrounded by cumulus cells. Right: seminiferous tubule cross-section. Leydig cells (blue), Sertoli cells (purple), basement membrane (pink), spermatogonia (green), spermatocytes (brown) and spermatids (pink and orange). ** denotes germ cell arrest gene for *FANCM* and *TEX14*. Genes were classified as unclear when they were not clearly linked to a specific organ.

The novel and established GDRs combined resulted in a total of 104 genes confidently linked to 120 human male infertility or abnormal genitourinary development phenotypes with moderate (n = 45), strong (n = 27) or definitive (n = 48) evidence (Tables  I and II). An overview of the organ or cell-level role(s) of these genes is detailed in Fig.  3. Another 138 GDRs were classified as ‘limited’ and are thus candidate genes whose dysfunction may result in a male infertility disorder (Supplementary Table SII). We propose that the latter (limited evidence) group will be of particular interest for review in the next few years.

**Table I:** Numbers of genes that are at least moderately linked to male infertility or abnormal genitourinary development phenotypes.

Description	AR	AD	XL	YL	Total
**Isolated infertility**	**27**	**5**	**4**	**0**	**36**
Acephalic sperm	3	0	0	0	3
Globozoospermia	1	0	0	0	1
Macrozoospermia	1	0	0	0	1
Multiple morphological abnormalities of the sperm flagella	13	1	0	0	14
Non-obstructive azoospermia or oligozoospermia	7	4	3	0	14
Congenital bilateral absence of the vas deferens	1	0	1	0	2
Fertilisation failure	1	0	0	0	1
**Syndromic infertility**	**13**	**3**	**1**	**0**	**17**
Primary ciliary dyskinesia	7	0	1	0	8
Other syndromes	6	3	0	0	9
**Endocrine disorder/Reproductive system syndrome**	**28**	**30**	**7**	**2**	**67**
Disorders of sexual development	14	13	4	2	33
Hypogonadotropic hypogonadism^*^	14	17	3	0	34

AR, autosomal recessive; AD, autosomal dominant; XL, X-linked; YL, Y-linked.

* We note that recent studies are identifying digenic/oligogenic causes for hypogonadotropic hypogonadism presentations.

**Table II:** List of genes linked to male infertility or abnormal genitourinary development phenotypes classified as moderate evidence or higher.

Gene	Location	Disorder	Inheritance pattern	Score	Conclusion
**Isolated infertility**					
***ADGRG2***	Xp22.13	Congenital bilateral absence of the vas deferens; OMIM:300985	XL	16	Definitive
***AR***	Xq12	Non-obstructive azoospermia; OMIM:NA	XL	17	Definitive
***ARMC2***	6q21	Multiple morphological abnormalities of the sperm flagella; OMIM:618433	AR	11	Moderate
***AURKC***	19q13.43	Macrozoospermia; OMIM:243060	AR	17	Definitive
***CFAP251***	12q24.31	Multiple morphological abnormalities of the sperm flagella; OMIM:NA (PS258150)	AR	17	Definitive
***CFAP43***	10q25.1	Multiple morphological abnormalities of the sperm flagella; OMIM:617592	AR	17	Definitive
***CFAP44***	3q13.2	Multiple morphological abnormalities of the sperm flagella; OMIM:617593	AR	17	Definitive
***CFAP65***	2q35	Multiple morphological abnormalities of the sperm flagella; OMIM:618664	AR	15	Strong
***CFAP69***	7q21.13	Multiple morphological abnormalities of the sperm flagella; OMIM:617959	AR	13	Strong
***CFAP91***	3q13.33	Multiple morphological abnormalities of the sperm flagella; OMIM:609910	AR	9	Moderate
***CFTR***	7q31.2	Congenital bilateral/unilateral absence of vas deferens; OMIM:277180	AR	17	Definitive
***DMRT1***	9p24.3	Non-obstructive azoospermia; OMIM:NA (PS258150)	AD	10	Moderate
***DNAH1***	3p21.1	Multiple morphological abnormalities of the sperm flagella; OMIM:617576	AR	17	Definitive
***DNAH17***	17q25.3	Multiple morphological abnormalities of the sperm flagella; OMIM:618643	AR	15	Strong
***DPY19L2***	12q14.2	Globozoospermia; OMIM:613958	AR	16	Definitive
***FANCM***	14q21.2	Oligozoospermia; OMIM:NA (PS258150)	AR	13	Strong
***FSIP2***	2q32.1	Multiple morphological abnormalities of the sperm flagella; OMIM:618153	AR	12	Moderate
***KLHL10***	17q21.2	Oligozoospermia; OMIM:615081	AD	10.5	Moderate
***M1AP***	2p13.1	Non-obstructive azoospermia; OMIM:619108	AR	12	Moderate
***MEI1***	22q13.2	Non-obstructive azoospermia; OMIM:NA (PS258150)	AR	13	Strong
***PLCZ1***	12p12.3	Fertilization failure; OMIM:617214	AR	16	Definitive
***PMFBP1***	16q22.2	Acephalic spermatozoa; OMIM:618112	AR	14	Strong
***QRICH2***	17q25.1	Multiple morphological abnormalities of the sperm flagella; OMIM:618341	AR	12	Moderate
***SEPTIN12***	16p13.3	Multiple morphological abnormalities of the sperm flagella; OMIM:614822	AD	11.5	Moderate
***SPEF2***	5p13.2	Multiple morphological abnormalities of the sperm flagella; OMIM:618751	AR	14.75	Strong
***STAG3***	7q22.1	Non-obstructive azoospermia; OMIM:NA (PS258150)	AR	11.5	Moderate
***SUN5***	20q11.21	Acephalic sperm; OMIM:617187	AR	16.75	Definitive
***SYCP2***	20q13.33	Severe oligozoospermia; OMIM:258150	AD	10.75	Moderate
***SYCP3***	12q23.2	Non-obstructive azoospermia; OMIM:270960	AD	14	Strong
***TEX11***	Xp11	Non-obstructive azoospermia; OMIM:309120	XL	16	Definitive
***TEX14***	17q22	Non-obstructive azoospermia; OMIM:617707	AR	10	Moderate
***TEX15***	8p12	Non-obstructive azoospermia; OMIM:617960	AR	13.5	Strong
***TSGA10***	2q11.2	Acephalic spermatozoa; OMIM:617961	AR	10.25	Moderate
***TTC29***	4q31.22	Multiple morphological abnormalities of the sperm flagella; OMIM:618745	AR	14.5	Strong
***USP26***	Xq26.2	Azoospermia or oligozoospermia; OMIM:NA (PS258150)	XL	9.5	Moderate
***XRCC2***	7q36.1	Non-obstructive azoospermia; OMIM: 617247	AR	10	Moderate
**Syndromic infertility**					
***APOA1***	11q23.3	Testicular amyloidosis; OMIM:105200	AD	12	Moderate
***CATSPER2***	15q15.3	Deafness infertility syndrome; OMIM: 611102	AR	11	Moderate
***CCDC39***	3q26.33	Primary ciliary dyskinesia; OMIM:613807	AR	13	Strong
***CCDC40***	17q25.3	Primary ciliary dyskinesia; OMIM:613808	AR	13.25	Strong
***CDC14A***	1p21.2	Oligoasthenoteratozoospermia OMIM:608653	AR	9	Moderate
***CEP290***	12q21.32	Leber congenital amaurosis; OMIM:611755	AR	9	Moderate
***DNAAF2***	14q21.3	Primary ciliary dyskinesia; OMIM:612518	AR	12.25	Moderate
***DNAAF4***	15q21.3	Primary ciliary dyskinesia; OMIM:615482	AR	13	Strong
***DNAAF6***	Xq22.3	Primary ciliary dyskinesia: OMIM:300991	XL	15	Strong
***FANCA***	16q24.3	Occult Fanconi anaemia; OMIM:NA (PS227650)	AR	10	Moderate
***LRRC6***	8q24.22	Primary ciliary dyskinesia; OMIM:614935	AR	13.5	Strong
***MNS1***	15q21.3	Asthenoteratozoospermia; OMIM:NA (PS258150)	AR	9.5	Moderate
***NLRP3***	1q44	Muckle-Wells Syndrome; OMIM:191900	AD	9	Moderate
***PKD1***	16p13.3	Polycystic kidney disease and asthenozoospermia; OMIM:173900	AD	11.25	Moderate
***RSPH3***	6q25.3	Primary ciliary dyskinesia; OMIM:616481	AR	10.25	Moderate
***SPEF2***	5p13.2	Primary ciliary dyskinesia with multiple morphological abnormalities of the sperm flagellum; OMIM:618751	AR	12	Moderate
***TRIM37***	17q22	Mulibrey nanism; OMIM:253250	AR	10	Moderate
**Reproductive system syndrome/endocrine disorder**					
***AMH***	19p13.3	Persistent Müllerian duct syndrome; OMIM:261550	AR	17	Definitive
***AMHR2***	12q13.13	Persistent Müllerian duct syndrome; OMIM:261550	AR	17	Definitive
***ANOS1***	Xp22.31	Kallmann syndrome; OMIM:308700	XL	16	Definitive
***ANOS1***	Xp22.31	Isolated hypogonadotropic hypogonadism (normosmic); OMIM:308700	XL	13	Strong
***AR***	Xq12	Partial androgen insensitivity syndrome; OMIM:312300/300633	XL	17	Definitive
***BMP4***	14q22.2	Hypospadias; OMIM:NA (PS300633). Micropenis; OMIM:NA	AD	10.25	Moderate
***BMP7***	20q13.31	Hypospadias; OMIM:NA (PS300633)	AD	10.25	Moderate
***BNC2***	9p22.3-p22.2	Hypospadias; OMIM:NA (PS300633)	AD	10	Moderate
***CCDC141***	2q31.2	Kallmann syndrome; OMIM:NA (PS147950)	AR	12	Moderate
***CHD7***	8q12.2	Kallmann syndrome without CHARGE phenotype; OMIM:612370	AD	16	Definitive
***CHD7***	8q12.2	Isolated hypogonadotropic hypogonadism (normosmic) without CHARGE phenotype; OMIM:612370	AD	17	Definitive
***CYP11A1***	15q24.1	Congenital adrenal insufficiency with partial 46,XY sex reversal (Prader stage 4; 5 or 6); OMIM:613743	AR	16	Definitive
***CYP11B1***	8q24.3	46,XX Disorders of sexual development (Prader scale 4; 5 or 6) due to congenital adrenal hyperplasia (11-beta-hydroxylase deficiency); OMIM: 202010	AR	17	Definitive
***CYP17A1***	10q24.32	46,XY Disorders of sexual development (Prader stage 4, 5 or 6) due to 17-alpha-hydroxylase/17,20-lyase deficiency; OMIM:202110	AR	16	Definitive
***CYP19A1***	15q21.2	Aromatase excess syndrome with gynaecomastia; OMIM:139300	AD	17	Definitive
***CYP19A1***	15q21.2	46,XX Disorders of sexual development (Prader scale 4; 5 or 6) due to aromatase deficiency; OMIM:613546	AR	16	Definitive
***CYP19A1***	15q21.2	Male infertility in 46,XY men due to aromatase deficiency; OMIM:613546	AR	9.5	Moderate
***CYP21A2***	6p21.33	Classic congenital adrenal hyperplasia; OMIM:201910	AR	17	Definitive
***CYP21A2***	6p21.33	Non-classic adrenal hyperplasia (late onset or no CAH symptoms); OMIM: 201910	AR	17	Definitive
***DHX37***	12q24.31	46,XY Disorders of sexual development (Prader scale 4; 5 or 6); OMIM:273250	AD	11	Moderate
***FGF17***	8p21.3	Kallmann syndrome; OMIM:615270	AD	9	Moderate
***FGF8***	10q24.32	Kallmann syndrome; OMIM: 612702	AD	10	Moderate
***FGF8***	10q24.32	Isolated hypogonadotropic hypogonadism (normosmic); OMIM:612702	AD	14	Strong
***FGFR1***	8p11.23	Kallmann syndrome; OMIM:147950	AD	17	Definitive
***FGFR1***	8p11.23	Isolated hypogonadotropic hypogonadism (normosmic); OMIM:147950	AD	17	Definitive
***FSHB***	11p14.1	Isolated hypogonadotropic hypogonadism; OMIM:229070	AR	12.25	Moderate
***FSHR***	2p16.3	Hypergonadotropic hypogonadism; OMIM:NA (PS147950)	AR	11	Moderate
***GATA4***	8p23.1	46,XY Disorders of sexual development (Prader scale 4; 5 or 6) resulting in anomalies of testicular development; OMIM:615542	AD	13	Strong
***GNRH1***	8p21.2	Isolated hypogonadotropic hypogonadism; OMIM:614841	AR	13.5	Strong
***GNRHR***	4q13.2	Isolated hypogonadotropic hypogonadism; OMIM:146110	AR	17	Definitive
***HS6ST1***	2q14.3	Kallmann syndrome; OMIM:614880	AD	9.5	Moderate
***HSD17B3***	9q22.32	46,XY Disorders of sexual development (Prader scale 4; 5 or 6) resulting in anomalies of testicular development; OMIM:264300	AR	16	Definitive
***HSD3B2***	1p12	Adrenal hyperplasia due to 3β-hydroxysteroid dehydrogenase deficiency; OMIM:201810	AR	16.5	Definitive
***IGSF10***	3q25.1	Delayed puberty; OMIM:NA (PS147950)	AD	9.25	Moderate
***IL17RD***	3p14.3	Kallmann syndrome with hearing loss; OMIM:615267	AD	14.5	Strong
***INSL3***	19p13.11	Cryptorchidism; OMIM:219050	AD	12	Moderate
***KISS1R***	19p13.3	Kallmann syndrome; OMIM:614837	AR	9	Moderate
***KISS1R***	19p13.3	Isolated hypogonadotropic hypogonadism (normosmic); OMIM:614837	AR	17	Definitive
***LHB***	19q13.33	Isolated Hypogonadotropic hypogonadism; OMIM:228300	AR	16.5	Definitive
***LHCGR***	2p16.3	Leydig cell dysfunction with hypogonadism; OMIM:238320	AR	16.5	Definitive
***LHCGR***	2p16.3	Male precocious puberty; OMIM:176410	AD	17	Definitive
***MAMLD1***	Xq28	46,XY Disorders of Sex Development (Prader scale 4; 5 or 6); OMIM:300758	XL	15	Strong
***MYRF***	11q12.2	46XY Disorders of Sex Development, OMIM gene 608329	AD	14.5	Strong
***NR0B1***	Xp21.2	Congenital adrenal hypoplasia; OMIM:300200	XL	17	Definitive
***NR0B1***	Xp21.2	Late-onset adrenal failure or isolated hypogonadotropic hypogonadism; OMIM:NA (PS147950)	XL	17	Definitive
***NR5A1***	9q33.3	46,XY Disorders of sexual development (Prader scale 4; 5 or 6); OMIM:612965	AD	17	Definitive
***NR5A1***	9q33.3	46,XX Disorders of sexual development (Prader scale 4; 5 or 6); OMIM:617480	AD	16	Definitive
***NR5A1***	9q33.3	Isolated spermatogenic failure; OMIM:184757	AD	14	Strong
***PLXNA1***	3q21.3	Kallmann syndrome; OMIM:NA (PS147950)	AD	13.5	Strong
***POU1F1***	3p11.2	Combined pituitary hormone deficiency; OMIM:613038	AR	16	Definitive
***PROK2***	3p13	Kallmann syndrome; OMIM:610628	AR	11.5	Moderate
***PROKR2***	20p12.3	Kallmann syndrome; OMIM:244200	AR	17	Definitive
***PROP1***	5q35.3	Pituitary hormone deficiency; OMIM:262600	AR	17	Definitive
***RSPO1***	1p34.3	Palmoplantar hyperkeratosis with squamous cell carcinoma of skin and sex reversal; OMIM:610644	AR	12	Moderate
***SEMA3A***	7q21.11	Kallmann syndrome; OMIM:614897	AD	16	Definitive
***SOX10***	22q13.1	Kallmann syndrome; OMIM:NA (PS147950)	AD	16	Definitive
***SOX2***	3q26.33	Isolated hypogonadotropic hypogonadism (normosmic); OMIM:NA (PS147950)	AD	16	Definitive
***SOX3***	Xq27.1	46,XX Disorders of sexual development (Prader scale 4; 5 or 6); OMIM:NA	XL	13	Strong
***SOX9***	17q24.3	46,XY Disorders of sexual development (Prader scale 4; 5 or 6); OMIM:NA	AD	13.5	Strong
***SRD5A2***	2p23.1	46,XY Disorders of sexual development (Prader scale 4; 5 or 6); OMIM:264600	AR	17	Definitive
***SRY***	Yp11.2	46,XX Disorders of sexual development (Prader scale 4; 5 or 6); OMIM:400045	YL	17	Definitive
***SRY***	Yp11.2	46,XY Disorders of sexual development (Prader scale 4; 5 or 6); OMIM:400044	YL	17	Definitive
***STAR***	8p11.23	Lipoid adrenal hyperplasia; OMIM:201710	AR	10	Moderate
***TACR3***	4q24	Kallmann syndrome; OMIM:614840	AR	16.5	Definitive
***WDR11***	10q26.12	Hypogonadotropic hypogonadism; OMIM:614858	AD	12	Moderate
***WDR11***	10q26.12	Kallmann syndrome; OMIM:614858	AD	11	Moderate
***WT1***	11p13	46,XY Disorders of sexual development (Prader scale 4; 5 or 6) without Wilm's tumour; OMIM:NA (PS400044)	AD	14.25	Strong

We note that recent studies are identifying digenic/oligogenic causes for hypogonadotropic hypogonadism presentations including Kallman syndrome.

### Insights into the genetic causes of male infertility

As outlined above, in recent years, the identification of genes confidently linked to a male infertility phenotype has risen by 150% from a stable average of approximately four per year in the period of 2000–2016 to 10 per year in the period of 2017–2019 (Fig.  2D). Additionally, the number of GDRs with limited (emerging) evidence is growing quickly, at an average of 17 new genes per year in the period 2017–2019 (Fig.  2D).

The majority of confident GDRs were endocrine disorders or reproductive system syndromes (n = 67) and isolated infertility phenotypes (n = 36), while a minority were linked to syndromic infertility (n = 17), including primary ciliary dyskinesia (PCD; Table  I). The systematic analysis of patients who present with multiple morphological abnormalities of the sperm flagella (MMAF) phenotypes was also of particular note. In 2018, there were five genes confidently linked to human MMAF. This has now tripled to a total of 15 genes as of mid-2020. Although representing only a small fraction of patients with isolated infertility, the use of NGS has the potential to diagnose up to ∼50% of MMAF patients (Toure *et al.*, 2021). A further 15 genes were confidently linked to the most frequently presenting clinical presentations, non-obstructive azoospermia (NOA) and oligozoospermia (Tables  I and II). The majority of all confident GDRs represented an autosomal recessive inheritance pattern (n = 68), while autosomal dominant (n = 38), X-linked (n = 12) and Y-linked (n = 2) inheritance patterns were also reported.

## Discussion

The aim of this study was to provide an updated assessment of all genetic variants reported as causative of human male infertility phenotypes. We also highlight male infertility phenotypes that have received significant attention in the last few years and identify a group of promising new candidate genes. These genes are currently classified as limited evidence, requiring additional replication studies and/or functional evidence in order to be classified as strongly associated with male infertility. Based on our findings, the authors/the IMIGC consortium recommend several updates to the genetic testing standards currently employed in the field of human male infertility, most important being the adoption of exome sequencing as the default sequencing approach.

Our clinical validity assessment revealed that as of 1 July 2020, a total of 104 genes were linked to a total of 120 male infertility or abnormal genitourinary development phenotypes. This is a significant increase from the 2019 report wherein 78 genes (33% increase to 104) were associated with 92 phenotypes (30% increase to 120) at a moderate or higher level of supporting evidence. As this previous assessment included all published reports from 1958 to 2018, this significant increase in 2 years further emphasises the healthy uptake of studies to elucidate genetic causes of male infertility, specifically those using NGS.

### Recent developments in genetics of male infertility research and diagnostics

The diagnostic rate of genetic tests for all types of isolated male infertility combined currently sits between 4% and 9.2% (Olesen *et al.*, 2017; Punab *et al.*, 2017; Tüttelmann *et al.*, 2018). These rates are notably behind levels seen in other heterogeneous disorders with a large genetic contribution such as developmental delay (∼30%) and cardiomyopathies (30–40%; Rehm, 2017). The biggest difference between these diseases is the slow uptake of NGS approaches in the male infertility field in research, but even more so in diagnostics. WES and WGS are now routinely being applied in the diagnostic follow-up of patients with other genetic disorders, resulting in the availability of very large cohorts for disease gene discovery (e.g. Bourinaris *et al.*, 2020; Cuvertino *et al.*, 2020; Kaplanis *et al.*, 2020). This, unfortunately, is still not the case for severe forms of male infertility in most countries. However, as highlighted in Fig.  2C and D, the field of male infertility genetics is now expanding rapidly, largely due to the reduced costs and increasing accessibility of NGS, which allows for a more complete and economic testing of patients. Consequently, the number of novel candidate genes and validated disease genes is rapidly growing, which is essential for further diagnostic implementation of these approaches in male infertility.

#### Multiple morphological abnormalities of the sperm flagellum

The phenotype where most progress has been made in recent years is MMAF. Of the 31 newly discovered genes confidently linked to male infertility, 9 were linked to MMAF (29%). When just considering genes likely associated with primary male infertility (i.e. spermatogenesis genes), this constitutes 47% of new, confidently classified genes (9 of 19). Currently, a total of 14 genes (*ARMC2*, *CFAP43*, *CFAP44*, *CFAP65*, *CFAP69*, *CFAP91*, *CFAP251*, *DNAH1*, *DNAH17*, *FSIP2*, *QRICH2*, *SEPTIN12*, *SPEF2 and TTC29*) are confidently linked to MMAF and another 9 (*AK7*, *AKAP4*, *CEP135*, *CFAP70*, *DNAH2*, *DNAH6*, *DZIP1, TTC21A and WDR19*) are listed as candidate genes, i.e. they have a limited classification. As a reflection of collaborative research efforts, MMAF-associated genes also comprise 42% of all confident genes that function during the spermiogenesis period.

Furthermore, and in line with the highly conserved core structure of motile cilia and flagella across tissues, there is a clear phenotypic continuum of patients with phenotypes ranging from classical PCD, manifesting as complete sperm immotility but normal cytology, to severe forms of teratozoospermia. Sperm tail development is a remarkable process that requires the expression of more than 1000 proteins (Toure *et al.*, 2021) and their co-ordinated transport into a distinct ciliary compartment originating from a modified centriole that docks to the sperm head (Pleuger *et al.*, 2020). Thus, there are likely many additional genes required for human/mammalian sperm tail development to be discovered. Novel evidence has been identified from MMAF studies, where variants in *SPEF2* cause PCD with MMAF (Tu *et al.*, 2020). While the origin of this commonality is largely unexplored, it may reflect shared protein transport pathways into the ciliary/sperm tail compartment (Pleuger *et al.*, 2020). There are also genes that play important roles in axoneme function (e.g. *DNAH17*; Whitfield *et al.*, 2019), whose loss of function results in an isolated infertility phenotype where cilia are unaffected.

#### Azoospermia and oligozoospermia

NOA and severe oligozoospermia are expected to have extensive genetic heterogeneity owing to the multiple phases of spermatogenesis that can be affected to cause these presentations. Although a few genes confidently linked to primary testicular failure are beginning to emerge, including *M1AP*, *STAG3*, *SYCP2 and TEX11*, where data from animal models indicate each is essential for meiosis (Yang *et al.*, 2006; Arango *et al.*, 2013; Winters *et al.*, 2014; Yatsenko *et al.*, 2015), large cohort sizes are critical to reveal the full spectrum of disease genes. The discovery and validation of three of these meiosis genes have been possible through the collaborative efforts of the IMIGC/GEMINI consortia and the use of large infertile cohorts and replication studies. This has now led to the availability of WES data for >3000 men with NOA or severe oligozoospermia, which underscores the importance of data sharing/collaboration for the continued identification of novel, rare genetic causes of male infertility. In addition, these large cohorts help to validate and better estimate the role of previously implicated variants/genes. For example, in 2017 variants in the D-box region of *PIWIL1* were implicated as a recurrent cause of NOA (Gou *et al.*, 2017). Using WES on 2740 men with NOA or severe oligozoospermia, the IMIGC/GEMINI consortia demonstrated that pathogenic variants in the D-box region of *PIWIL1* specifically, and variants elsewhere in the gene, are not a common cause of male infertility (Oud *et al.*, 2021).

### Identifying the variant(s) causative for male infertility

Currently, ∼33% of all identified GDRs proposed in the literature fell into the ‘unable to classify’ category. For most, this classification is given because the inheritance pattern is unclear. The list, however, likely contains multiple genes that play an important role in the aetiology of monogenic male infertility. As with any disease, but perhaps exacerbated by the wide range of individual biological processes required for male fertility, it can be difficult to determine the role of poorly characterised genes, and whether loss of their function results in a disease state. To assist in filtering for high confidence variants, members of the GEMINI consortium have developed the ‘Population Sampling Probability’ pipeline (PSAP; Wilfert *et al.*, 2016). This is particularly useful when it is the first time a damaging variant has been found in a novel gene, where calculations are based on inheritance model (autosomal dominant, recessive, etc.) and allele frequency. As has been highlighted by the GEMINI consortium, this tool is useful for discovering promising new candidate disease genes and individual pathogenic variants, but independent replication and functional validation is still critically important before genes can be used in a diagnostic setting as a ‘validated disease gene’.

### Dynamics of genotype–phenotype mapping

Recent genetic male infertility studies suggest that a high percentage of diagnoses can be revealed, especially in cohorts of men with maturation arrest. A clear determination of the point of arrest (i.e. at spermatogonia or spermatocytes) can assist in refining possibly relevant genes containing variants, based on any known biological function(s). However, very little so far has been done outside of meiotic and spermatogonial arrest. Various genes have also been linked to multiple phenotypic outcomes; thus, the establishment of a clear genotype–phenotype correlation is not necessarily straightforward. One such example is the *TEX14* gene, where pathogenic variation can lead to a Sertoli cell-only phenotype or spermatocyte arrest (Fakhro *et al.*, 2018). The progressive loss of germ cells may occur as a factor of age owing to a role in spermatogonial stem cell renewal, as suggested for *Nxf2* knockout mice (Pan *et al.*, 2009). Alternatively, environmental exposures may interact with the genotype to lead to an exacerbation of the phenotype. Variability in the genotype–phenotype relationship has also been identified for single gene variants in reproductive system syndromes/endocrine disorders (Domenice *et al.*, 2016; Goncalves *et al.*, 2017) in 12 of the 13 (92%) confident genes—*ANOS1*, *AR*, *CHD7*, *CYP19A1*, *CYP21A2*, *FGF8*, *FGFR1*, *KISS1R*, *LHCGR*, *NR0B1*, *NR5A1 and WDR11*. These disorders are largely complex in that they effect multiple hormonal pathways and tissues beyond the testis.

Other presentations with variability in genotype and phenotype include congenital hypogonadotropic hypogonadism (HH)/Kallmann syndrome. In addition to Mendelian causes, incomplete penetrance and oligogenic forms are thought to be causative for these disorders (Maione *et al.*, 2018). One example is the *PROK2* gene, for which autosomal dominant, recessive and oligogenicity have been proposed to be causative (Leroy *et al.*, 2008). For the DSD spectrum and HH, research groups are now actively exploring the role of oligogenic inheritance in contributing to the disease (Cangiano *et al.*, 2021). For DSDs, this genetic explanation has been proposed following the finding that many patients present with at least one variant in two individual known or novel DSD genes, particularly when *MAMLD1* is affected (Camats *et al.*, 2020; Li *et al.*, 2020). Similarly, variants in *FGFR1* and other HH genes have been shown to cause Kallman syndrome in an oligogenic manner (reviewed in Cangiano *et al.*, 2021).

### Recommendations for genetic testing in male infertility

Although NGS methods are frequently used in research laboratories to study the genetics of male infertility, they have not yet been extensively employed in clinical genetic diagnostics for this condition. While there are no standardised international guidelines for clinical genetic testing in male infertility, a small number of guidelines have been published by professional andrology groups. Examples include guidelines for oligoasthenoteratozoospermia (OAT; Colpi *et al.*, 2018) and Klinefelter syndrome (Zitzmann *et al.*, 2021) by the European Academy of Andrology, and diagnostics/treatment prior to use of ART (Toth *et al.*, 2019) by the German Association of the Scientific Medical Societies. However, sperm count cut-offs and genetic testing approaches vary widely between countries. Patients with oligozoospermia, <5 million sperm/ml (EU/USA), or azoospermia are generally offered karyotyping and Y-chromosome microdeletion analysis, while *CFTR* testing is recommended for men with suspected congenital bilateral absence of the vas deferens (Krausz and Riera-Escamilla, 2018). These guidelines, or in some cases purely expert opinions, thus limit the genetic testing to the most common infertility presentations and exclude patients with other sperm phenotypes, such as teratozoospermia/asthenoteratozoospermia (e.g. globozoospermia and MMAF), and other sperm motility disorders (asthenozoospermia/OAT). According to the average clinical presentation of German men over the last 30 years, this means that at least 40% of infertile men are not being offered any form of genetic testing (Tüttelmann *et al.*, 2018).

In line with the definitive evidence that pathogenic variants in at least 36 genes may result in isolated infertility, we advocate for an update of genetic testing guidelines for male infertility. Ideally, the blood of infertile men should be used for WES to identify any protein-coding variants in a non-biased manner. We appreciate there will often be an issue with a lack of expertise in this technology, or access to sequencing platforms. IMIGC members (imigc.org) are situated across the USA, Europe and Australia, and are interested in forming additional collaborations for clinical and research purposes. As a less impactful, backup approach, the genetic targets of panels used in targeting sequencing should at least be updated to include the genes validated in this study (those moderately linked and stronger). Additionally, a wider subset of patients/phenotypes should be sequenced. Such approaches will help to achieve more diagnoses, better-personalised treatment, improved risk assessment for the transmission of infertility to offspring and better counselling for potential health risks in the infertile man. It will also help to improve counselling of azoospermic men prior to testicular biopsy, as finding a genetic diagnosis may help in predicting the chances for successful testicular sperm extraction, which is an incredibly invasive procedure.

### Wider implications of male infertility genetics

Precise phenotyping of men with pathogenic variants will assist in evaluating potential future health risks. Mounting evidence suggests that male infertility may pose as a predictive condition for life-threatening comorbidities in later life, such as various types of cancer and cardiovascular disease (Jensen *et al.*, 2009; Eisenberg *et al.*, 2014; Ehrlich, 2015). While the precise mechanism(s) underpinning this observation are unknown, it is notable that many genes that are highly expressed in the testis and lead to male infertility when mutated are also expressed in other organs, but at considerably lower levels. These include, for example, multiple DNA repair genes linked to infertility and various cancers (reviewed in Nagirnaja *et al.*, 2018) and *TEX11*, which is expressed in the testis and the pancreas according to RNA-seq data (GTEx Portal). As has been made clear above, this is the case for several cilia genes, where men often present with chronic bronchitis and other lung conditions (reviewed in Leigh *et al.*, 2009; Sironen *et al.*, 2020). Thus, additional studies where the health of infertile men is broadly investigated are urgently needed.

Although the topic could constitute an entire article itself, the rapidly increasing use of ART worldwide (de Mouzon *et al.*, 2020) is resulting in the propagation of infertility-causing variants throughout the population. As we identify these variants in men who undergo ART, we will also be faced with delivering this information back to the patients through genetic counselling services. This is especially relevant for men with Y-linked variants, where any sons will presumably be inflicted with the same infertility phenotype as their affected fathers and thus also require ART if wanting their own biological child.

### Strengths and limitations

We used the joint expertise of the IMIGC to systematically evaluate all available evidence (as of 1 July 2020) for monogenic causes of isolated or syndromic male infertility, endocrine disorders or reproductive system abnormalities affecting the male sex organs. No other published reviews in this field come close to including the ∼600 genes comprehensively evaluated here. We improved the quality of evidence scoring by including a larger number of reviewers from independent research groups with broad expertise in male infertility. As all research groups have been involved in disease gene discovery of various genes described in this study, we prevented bias as much as possible by not allowing scoring by reviewers who have worked on respective genes.

## Conclusion

Here, we describe an updated clinical validity assessment for a total of 657 male infertility or abnormal genitourinary development GDRs, involving 596 genes. We identified 104 genes linked to 120 phenotypes with sufficient evidence for use in gene panels. These results may help to improve genetic testing in male infertility research and/or diagnostics.

## Supplementary data

Supplementary data are available at *Human Reproduction Update* online.

## Data availability

The present study and the corresponding search protocol were registered with the PROSPERO registry (http://www.crd.york.ac.uk/PROSPERO) as PROSPERO 2021: CRD42021229164. All scoring spreadsheets are accessible via the IMIGC website (www.imigc.org/data-sharing).

## Supplementary Material

dmab030_Supplementary_Data

## References

[dmab030-B1] Alhathal N , Maddirevula S, Coskun S, Alali H, Assoum M, Morris T, Deek HA, Hamed SA, Alsuhaibani S, Mirdawi A et al A genomics approach to male infertility. Genet Med 2020;22:1967–1975. 32719396 10.1038/s41436-020-0916-0

[dmab030-B2] Arafat M , Harlev A, Har-Vardi I, Levitas E, Priel T, Gershoni M, Searby C, Sheffield VC, Lunenfeld E, Parvari R. Mutation in *CATIP* (C2orf62) causes oligoteratoasthenozoospermia by affecting actin dynamics. J Med Genet 2020. doi:10.1136/jmedgenet-2019-106825. 10.1136/jmedgenet-2019-106825 32503832

[dmab030-B3] Arango NA , Li L, Dabir D, Nicolau F, Pieretti-Vanmarcke R, Koehler C, McCarrey JR, Lu N, Donahoe PK. Meiosis I arrest abnormalities lead to severe oligozoospermia in meiosis 1 arresting protein (*M1ap*)-deficient mice. Biol Reprod 2013;88:76. 23269666 10.1095/biolreprod.111.098673 PMC4013860

[dmab030-B4] Bedoni N , Haer-Wigman L, Vaclavik V, Tran VH, Farinelli P, Balzano S, Royer-Bertrand B, El-Asrag ME, Bonny O, Ikonomidis C et al Mutations in the polyglutamylase gene *TTLL5*, expressed in photoreceptor cells and spermatozoa, are associated with cone-rod degeneration and reduced male fertility. Hum Mol Genet 2016;25:4546–4555. 28173158 10.1093/hmg/ddw282

[dmab030-B5] Bourinaris T , Smedley D, Cipriani V, Sheikh I, Athanasiou-Fragkouli A, Chinnery P, Morris H, Real R, Harrison V, Reid E et al; Genomics England Research Consortium. Identification of *UBAP1* mutations in juvenile hereditary spastic paraplegia in the 100,000 Genomes Project. Eur J Hum Genet 2020;28:1763–1768. 32934340 10.1038/s41431-020-00720-w PMC7784862

[dmab030-B6] Camats N , Fluck CE, Audi L. Oligogenic origin of differences of sex development in humans. Int J Mol Sci 2020;21: 1809. 10.3390/ijms21051809 PMC7084473 32155719

[dmab030-B7] Cangiano B , Swee DS, Quinton R, Bonomi M. Genetics of congenital hypogonadotropic hypogonadism: peculiarities and phenotype of an oligogenic disease. Hum Genet 2021;140:77–111. 32200437 10.1007/s00439-020-02147-1

[dmab030-B8] Chen S , Wang G, Zheng X, Ge S, Dai Y, Ping P, Chen X, Liu G, Zhang J, Yang Y et al Whole-exome sequencing of a large Chinese azoospermia and severe oligospermia cohort identifies novel infertility causative variants and genes. Hum Mol Genet 2020;29:2451–2459. 32469048 10.1093/hmg/ddaa101

[dmab030-B9] Chiu W , Hsun YH, Chang KJ, Yarmishyn AA, Hsiao YJ, Chien Y, Chien CS, Ma C, Yang YP, Tsai PH et al Current genetic survey and potential gene-targeting therapeutics for neuromuscular diseases. Int J Mol Sci 2020;21: 9589. 10.3390/ijms21249589 PMC7767109 33339321

[dmab030-B10] Colpi GM , Francavilla S, Haidl G, Link K, Behre HM, Goulis DG, Krausz C, Giwercman A. European Academy of Andrology guideline Management of oligo-astheno-teratozoospermia. Andrology 2018;6:513–524. 30134082 10.1111/andr.12502

[dmab030-B2854108] Coutton C, , Martinez G, , Kherraf Z-E, , Amiri-Yekta A, , Boguenet M, , Saut A, , He X, , Zhang F, , Cristou-Kent M, , Escoffier J et al Bi-allelic Mutations in ARMC2 Lead to Severe Astheno-Teratozoospermia Due to Sperm Flagellum Malformations in Humans and Mice. *Am J Hum Genet* 2019;104:331–340. 30686508 10.1016/j.ajhg.2018.12.013 PMC6372258

[dmab030-B11] Cuvertino S , Hartill V, Colyer A, Garner T, Nair N, Al-Gazali L, Canham N, Faundes V, Flinter F, Hertecant J et al; Genomics England Research Consortium. A restricted spectrum of missense *KMT2D* variants cause a multiple malformations disorder distinct from Kabuki syndrome. Genet Med 2020;22:867–877. 31949313 10.1038/s41436-019-0743-3 PMC7200597

[dmab030-B12] de Mouzon J , Chambers GM, Zegers-Hochschild F, Mansour R, Ishihara O, Banker M, Dyer S, Kupka M, Adamson GD. International Committee for Monitoring Assisted Reproductive Technologies world report: assisted reproductive technology 2012. Hum Reprod 2020;35:1900–1913. 32699900 10.1093/humrep/deaa090

[dmab030-B13] Domenice S , Machado AZ, Ferreira FM, Ferraz-de-Souza B, Lerario AM, Lin L, Nishi MY, Gomes NL, da Silva TE, Silva RB et al Wide spectrum of *NR5A1*-related phenotypes in 46,XY and 46,XX individuals. Birth Defects Res C Embryo Today 2016;108:309–320. 28033660 10.1002/bdrc.21145 PMC5347970

[dmab030-B14] Ehrlich S. Effect of fertility and infertility on longevity. Fertil Steril 2015;103:1129–1135. 25934598 10.1016/j.fertnstert.2015.03.021

[dmab030-B15] Eisenberg ML , Li S, Behr B, Cullen MR, Galusha D, Lamb DJ, Lipshultz LI. Semen quality, infertility and mortality in the USA. Hum Reprod 2014;29:1567–1574. 24838701 10.1093/humrep/deu106 PMC4059337

[dmab030-B16] Fagerberg L , Hallstrom BM, Oksvold P, Kampf C, Djureinovic D, Odeberg J, Habuka M, Tahmasebpoor S, Danielsson A, Edlund K et al Analysis of the human tissue-specific expression by genome-wide integration of transcriptomics and antibody-based proteomics. Mol Cell Proteomics 2014;13:397–406. 24309898 10.1074/mcp.M113.035600 PMC3916642

[dmab030-B17] Fakhro KA , Elbardisi H, Arafa M, Robay A, Rodriguez-Flores JL, Al-Shakaki A, Syed N, Mezey JG, Abi Khalil C, Malek JA et al Point-of-care whole-exome sequencing of idiopathic male infertility. Genet Med 2018;20:1365–1373. 29790874 10.1038/gim.2018.10

[dmab030-B18] Goncalves CI , Fonseca F, Borges T, Cunha F, Lemos MC. Expanding the genetic spectrum of *ANOS1* mutations in patients with congenital hypogonadotropic hypogonadism. Hum Reprod 2017;32:704–711. 28122887 10.1093/humrep/dew354

[dmab030-B19] Gou LT , Kang JY, Dai P, Wang X, Li F, Zhao S, Zhang M, Hua MM, Lu Y, Zhu Y et al Ubiquitination-deficient mutations in human *Piwi* cause male infertility by impairing histone-to-protamine exchange during spermiogenesis. Cell 2017;169:1090–1104.e13. 28552346 10.1016/j.cell.2017.04.034 PMC5985145

[dmab030-B20] Jensen TK , Jacobsen R, Christensen K, Nielsen NC, Bostofte E. Good semen quality and life expectancy: a cohort study of 43,277 men. Am J Epidemiol 2009;170:559–565. 19635736 10.1093/aje/kwp168

[dmab030-B21] Kaplanis J , Samocha KE, Wiel L, Zhang Z, Arvai KJ, Eberhardt RY, Gallone G, Lelieveld SH, Martin HC, McRae JF et al; Deciphering Developmental Disorders Study. Evidence for 28 genetic disorders discovered by combining healthcare and research data. Nature 2020;586:757–762. 33057194 10.1038/s41586-020-2832-5 PMC7116826

[dmab030-B22] Krausz C , Riera-Escamilla A. Genetics of male infertility. Nat Rev Urol 2018;15:369–384. 29622783 10.1038/s41585-018-0003-3

[dmab030-B23] Leigh MW , Pittman JE, Carson JL, Ferkol TW, Dell SD, Davis SD, Knowles MR, Zariwala MA. Clinical and genetic aspects of primary ciliary dyskinesia/Kartagener syndrome. Genet Med 2009;11:473–487. 19606528 10.1097/GIM.0b013e3181a53562 PMC3739704

[dmab030-B24] Leroy C , Fouveaut C, Leclercq S, Jacquemont S, Boullay HD, Lespinasse J, Delpech M, Dupont JM, Hardelin JP, Dode C. Biallelic mutations in the prokineticin-2 gene in two sporadic cases of Kallmann syndrome. Eur J Hum Genet 2008;16:865–868. 18285834 10.1038/ejhg.2008.15

[dmab030-B25] Li L , Gao F, Fan L, Su C, Liang X, Gong C. Disorders of sex development in individuals harbouring *MAMLD1* variants: WES and interactome evidence of oligogenic inheritance. Front Endocrinol (Lausanne) 2020;11:582516. 33424767 10.3389/fendo.2020.582516 PMC7786837

[dmab030-B26] Liu C , Tu C, Wang L, Wu H, Houston BJ, Mastrorosa FK, Zhang W, Shen Y, Wang J, Tian S et al Deleterious variants in X-linked *CFAP47* induce asthenoteratozoospermia and primary male infertility. Am J Hum Genet 2021;108:309–323. 33472045 10.1016/j.ajhg.2021.01.002 PMC7895902

[dmab030-B27] Maione L , Dwyer AA, Francou B, Guiochon-Mantel A, Binart N, Bouligand J, Young J. GENETICS IN ENDOCRINOLOGY: genetic counseling for congenital hypogonadotropic hypogonadism and Kallmann syndrome: new challenges in the era of oligogenism and next-generation sequencing. Eur J Endocrinol 2018;178:R55–R80. 29330225 10.1530/EJE-17-0749

[dmab030-B28] Markitantova Y , Simirskii V. Inherited eye diseases with retinal manifestations through the eyes of homeobox genes. Int J Mol Sci 2020;21: 1602. 10.3390/ijms21051602 PMC7084737 32111086

[dmab030-B29] Nagirnaja L , Aston KI, Conrad DF. Genetic intersection of male infertility and cancer. Fertil Steril 2018;109:20–26. 29307395 10.1016/j.fertnstert.2017.10.028 PMC5761685

[dmab030-B30] Olesen IA , Andersson AM, Aksglaede L, Skakkebaek NE, Rajpert-de Meyts E, Joergensen N, Juul A. Clinical, genetic, biochemical, and testicular biopsy findings among 1,213 men evaluated for infertility. Fertil Steril 2017;107:74–82.e7. 27793385 10.1016/j.fertnstert.2016.09.015

[dmab030-B31] Oud MS , Volozonoka L, Friedrich C, Kliesch S, Nagirnaja L, Gilissen C, O'Bryan MK, McLachlan RI, Aston KI, Tuttelmann F et al Lack of evidence for a role of *PIWIL1* variants in human male infertility. Cell 2021;184:1941–1942. 33861957 10.1016/j.cell.2021.03.001 PMC7614813

[dmab030-B32] Oud MS , Volozonoka L, Smits RM, Vissers L, Ramos L, Veltman JA. A systematic review and standardized clinical validity assessment of male infertility genes. Hum Reprod 2019;34:932–941. 30865283 10.1093/humrep/dez022 PMC6505449

[dmab030-B33] Pan J , Eckardt S, Leu NA, Buffone MG, Zhou J, Gerton GL, McLaughlin KJ, Wang PJ. Inactivation of *Nxf2* causes defects in male meiosis and age-dependent depletion of spermatogonia. Dev Biol 2009;330:167–174. 19345203 10.1016/j.ydbio.2009.03.022 PMC2702087

[dmab030-B34] Pleuger C , Lehti MS, Dunleavy JE, Fietz D, O'Bryan MK. Haploid male germ cells-the Grand Central Station of protein transport. Hum Reprod Update 2020;26:474–500. 32318721 10.1093/humupd/dmaa004

[dmab030-B35] Punab M , Poolamets O, Paju P, Vihljajev V, Pomm K, Ladva R, Korrovits P, Laan M. Causes of male infertility: a 9-year prospective monocentre study on 1737 patients with reduced total sperm counts. Hum Reprod 2017;32:18–31. 27864361 10.1093/humrep/dew284 PMC5165077

[dmab030-B36] Rehm HL. Evolving health care through personal genomics. Nat Rev Genet 2017;18:259–267. 28138143 10.1038/nrg.2016.162 PMC6517837

[dmab030-B37] Schultz N , Hamra FK, Garbers DL. A multitude of genes expressed solely in meiotic or postmeiotic spermatogenic cells offers a myriad of contraceptive targets. Proc Natl Acad Sci U S A 2003;100:12201–12206. 14526100 10.1073/pnas.1635054100 PMC218736

[dmab030-B38] Sironen A , Shoemark A, Patel M, Loebinger MR, Mitchison HM. Sperm defects in primary ciliary dyskinesia and related causes of male infertility. Cell Mol Life Sci 2020;77:2029–2048. 31781811 10.1007/s00018-019-03389-7 PMC7256033

[dmab030-B39] Smith ED , Radtke K, Rossi M, Shinde DN, Darabi S, El-Khechen D, Powis Z, Helbig K, Waller K, Grange DK et al Classification of genes: standardized clinical validity assessment of gene-disease associations aids diagnostic exome analysis and reclassifications. Hum Mutat 2017;38:600–608. 28106320 10.1002/humu.23183 PMC5655771

[dmab030-B40] Toth B , Baston-Bust DM, Behre HM, Bielfeld A, Bohlmann M, Buhling K, Dittrich R, Goeckenjan M, Hancke K, Kliesch S et al Diagnosis and treatment before assisted reproductive treatments. guideline of the DGGG, OEGGG and SGGG (S2k Level, AWMF register number 015-085, February 2019)—part 2, hemostaseology, andrology, genetics and history of malignant disease. Geburtshilfe Frauenheilkd 2019;79:1293–1308. 31875859 10.1055/a-1017-3478 PMC6924330

[dmab030-B41] Toure A , Martinez G, Kherraf ZE, Cazin C, Beurois J, Arnoult C, Ray PF, Coutton C. The genetic architecture of morphological abnormalities of the sperm tail. Hum Genet 2021;140:21–42. 31950240 10.1007/s00439-020-02113-x

[dmab030-B42] Tu C , Nie H, Meng L, Wang W, Li H, Yuan S, Cheng D, He W, Liu G, Du J et al Novel mutations in *SPEF2* causing different defects between flagella and cilia bridge: the phenotypic link between MMAF and PCD. Hum Genet 2020;139:257–271. 31942643 10.1007/s00439-020-02110-0

[dmab030-B43] Tüttelmann F , Ruckert C, Ropke A. Disorders of spermatogenesis: perspectives for novel genetic diagnostics after 20 years of unchanged routine. Med Genet 2018;30:12–20. 29527098 10.1007/s11825-018-0181-7 PMC5838132

[dmab030-B44] Uhlen M , Fagerberg L, Hallstrom BM, Lindskog C, Oksvold P, Mardinoglu A, Sivertsson A, Kampf C, Sjostedt E, Asplund A et al Proteomics. Tissue-based map of the human proteome. Science 2015;347:1260419. 25613900 10.1126/science.1260419

[dmab030-B45] Wang X , Jin H, Han F, Cui Y, Chen J, Yang C, Zhu P, Wang W, Jiao G, Wang W et al Homozygous *DNAH1* frameshift mutation causes multiple morphological anomalies of the sperm flagella in Chinese. Clin Genet 2017;91:313–321. 27573432 10.1111/cge.12857

[dmab030-B46] Whatley M , Francis A, Ng ZY, Khoh XE, Atlas MD, Dilley RJ, Wong EYM. Usher syndrome: genetics and molecular links of hearing loss and directions for therapy. Front Genet 2020;11:565216. 33193648 10.3389/fgene.2020.565216 PMC7642844

[dmab030-B47] Whitfield M , Thomas L, Bequignon E, Schmitt A, Stouvenel L, Montantin G, Tissier S, Duquesnoy P, Copin B, Chantot S et al Mutations in *DNAH17*, encoding a sperm-specific axonemal outer dynein arm heavy chain, cause isolated male infertility due to asthenozoospermia. Am J Hum Genet 2019;105:198–212. 31178125 10.1016/j.ajhg.2019.04.015 PMC6612517

[dmab030-B48] Wilfert AB , Chao KR, Kaushal M, Jain S, Zollner S, Adams DR, Conrad DF. Genome-wide significance testing of variation from single case exomes. Nat Genet 2016;48:1455–1461. 27776118 10.1038/ng.3697 PMC5127779

[dmab030-B49] Winters T , McNicoll F, Jessberger R. Meiotic cohesin *STAG3* is required for chromosome axis formation and sister chromatid cohesion. EMBO J 2014;33:1256–1270. 24797474 10.1002/embj.201387330 PMC4198028

[dmab030-B50] Yang F , De La Fuente R, Leu NA, Baumann C, McLaughlin KJ, Wang PJ. Mouse *SYCP2* is required for synaptonemal complex assembly and chromosomal synapsis during male meiosis. J Cell Biol 2006;173:497–507. 16717126 10.1083/jcb.200603063 PMC2063860

[dmab030-B51] Yatsenko AN , Georgiadis AP, Ropke A, Berman AJ, Jaffe T, Olszewska M, Westernstroer B, Sanfilippo J, Kurpisz M, Rajkovic A et al X-linked *TEX11* mutations, meiotic arrest, and azoospermia in infertile men. N Engl J Med 2015;372:2097–2107. 25970010 10.1056/NEJMoa1406192 PMC4470617

[dmab030-B52] Yuan P , Yang C, Ren Y, Yan J, Nie Y, Yan L, Qiao J. A novel homozygous mutation of phospholipase C zeta leading to defective human oocyte activation and fertilization failure. Hum Reprod 2020;35:977–985. 32142120 10.1093/humrep/dez293

[dmab030-B53] Zitzmann M , Aksglaede L, Corona G, Isidori AM, Juul A, T'Sjoen G, Kliesch S, D'Hauwers K, Toppari J, Słowikowska-Hilczer J et al European academy of andrology guidelines on Klinefelter Syndrome Endorsing Organization: European Society of Endocrinology. Andrology 2021;9:145–167. 32959490 10.1111/andr.12909

